# Recent insights on phage therapy against multidrug-resistant *Acinetobacter baumannii*

**DOI:** 10.1186/s13568-025-01837-1

**Published:** 2025-03-12

**Authors:** Ann A. Elshamy, Sandra K. Kamal, Mariam T. Mahmoud, Aya M. Elhasany, Aya A. Shady, Sherok A. Mohamed, Hasna A. Abd-Elmaaboud, Nour E. El-Awady, Rana A. Mohamed, Sara A. El-Mirghany, Sarraa W. El-Hady, Mohamed M. Abd-ElRahman, Khaled M. Aboshanab

**Affiliations:** 1 Department of Microbiology and Immunology, Faculty of Pharmacy, Ain Shams University, Cairo, 11566 Egypt; 2 Faculty of Pharmacy, Ain Shams University, Cairo, 11566 Egypt

**Keywords:** *Acinetobacter baumannii*, Phage therapy, Antimicrobial resistance, Multidrug resistance, Phage cocktail

## Abstract

*Acinetobacter baumannii* is a prevalent clinical pathogen commonly found to be multidrug-resistant (MDR), causing serious to life-threatening infections, particularly hospital-acquired infections with limited therapeutic options. The MDR phenotype developed against this critical pathogen is increasingly developed globally, reaching a pan-drug-resistant phenotype conferring non-susceptibility to all antimicrobials used in its treatment according to the standard guidelines. Therefore, it is critical to develop innovative treatment approaches, such as phage therapy, considering the rise in drug-resistant *A. baumannii* infections. In this review, we highlight and discuss the up-to-date antimicrobial resistance of *A. baumannii*, the use of phages, their limitations, and future perspectives in treating *A. baumannii* infections. In addition, the combination of phages with antimicrobials, preclinical and clinical studies including pharmacokinetics and pharmacodynamics properties have been discussed.

## Introduction

The global misuse and overuse of antibiotics have led to an increased level of prevalence of highly resistant bacteria which have adapted structurally and genomically to overcome the effects of antibiotics used either for empiric or susceptibility test-based treatment (Moubareck and Hammoudi Halat 2020). These resistant bacteria have minimized humanity’s defense lines to include an extremely narrow range of therapeutic agents (Moubareck and Hammoudi Halat 2020). Despite the introduction of various new antimicrobial agents to our treatment options, resistance has become an ever-expanding concern, evolving at a geometric pace (Kyriakidis et al. 2021). This troubling trend indicates that, rather than diminishing, the problem of antibiotic resistance is growing increasingly complex (Kyriakidis et al. 2021). As we work to combat bacterial infections, the rising prevalence of resistance significantly complicates our efforts, highlighting the critical need for continued research and innovative approaches in antimicrobial strategies (Kyriakidis et al. 2021).

One of the most resilient bacteria that has experienced genomic and structural adaptations over the past 3 decades is the Gram-negative Genus *Acinetobacter* spp. Among its species, *baumannii* has risen to prominence as one of the most persistent pathogens faced, according to the World Health Organization (WHO) (Talebi Bezmin Abadi et al. 2019). One of the ESKAPE pathogens that threaten public health globally because of their emerging and ever-increasing resistance is *A. baumannii* (Kyriakidis et al. 2021). Its clinical relevance has surged over the last 15 years, largely due to its exceptional capacity to enhance resistance factors, positioning it as a significant threat in the current era of antibiotics. There have been reports of *A. baumannii* isolates showing resistance against every single class of potent antimicrobials in use, representing a critical situation that demands immediate attention from the global healthcare community (Mabrouk et al. 2020; Larsson and Flach 2022; Selim et al. 2024).

Recent studies have confirmed the role of transferrable plasmids conferring resistance to very important and effective classes of antibiotics such as carbapenems (Gutiérrez et al. 2024; Selim et al. 2024), tigecycline (Jia et al. 2025), fluoroquinolones (Hamed et al. 2018; Mohammed et al. 2021), and colistin (Martins-Sorenson et al. 2020) suggesting the possibility of outbreaks in clinical settings. In addition to the possibility and dissemination of acquiring extensively drug-resistant (XDR) and pandrug-resistant (PDR) phenotypes among clinical isolates of *A. baumannii* posing therapeutic challenges (Mabrouk et al. 2020). Previous studies have confirmed the correlation between acquiring antibiotic-resistance genes and the evolution of multidrug-resistant (MDR) phenotype among Gram-negative bacterial pathogens, particularly *A. baumannii* (Abdelaziz et al. 2021; Kousovista et al. 2021).

## The epidemiology and risk factors associated with *A. baumannii* infections

*A. baumannii* can cause both community-acquired and hospital-acquired infections, although the latter is the most common form (Fournier et al. 2006). The main risk factors associated with *A. baumannii* infections include prolonged hospitalization, previous use of broad-spectrum antibiotics, invasive procedures, and preexisting conditions or chronic diseases (Benaissa et al. 2023; Ibrahim, 2019). Hospital-acquired *Acinetobacter* infections are often device-associated, including ventilator-associated pneumonia (VAP) due to mechanical ventilation and prolonged oxygen sessions, catheter-associated urinary tract infections, and sepsis due to surgical and medical tools (Rangel and De-Simone 2024; Weinberg et al. 2020). The prevalence of MDR strains in patients with VAP was found to be 56.5% in Argentina, 61.8% in Taiwan, and 100% in Central America, Lebanon, Croatia, Pakistan, and Qatar, while the mortality rate was as high as 56.2% (Mohd Sazlly Lim et al. 2019). High incidence rates of carbapenem resistance among *A. baumannii* isolates have been reported in Europe as well as in Lebanon, Syria, Iraq, and Jordan (Kyriakidis et al. 2021). To combat such spread, strict sterilization measures should be implemented in all medical facilities, especially in the treatment of chronically diseased patients such as diabetics, cancer patients, and chronic obstructive pulmonary disease (COPD) patients, which have shown more susceptibility to the previously mentioned pathogen (Maragakis and Perl 2008).

## The mechanisms of resistance of *A. baumannii* to antibiotics

MDR strains of *A. baumannii* have been reported, and there have been multiple cases of the bacteria rapidly becoming resistant to antibiotics. Many strains of *A. baumannii* are now highly resistant to most of the antibiotics that are currently available on the market (Lee et al. 2017; Lin and Lan 2014). *A. baumannii* has developed a range of resistance mechanisms: (i) efflux pumps which expel antibiotics from the cell leading to resistance against several different classes of antibiotics by preventing access to the target; (ii) β-lactamases which significantly antagonize the effectiveness of β-lactam antibiotics such as penicillins, cephalosporins, monobactams, carbapenems and cephalosporins; (iii) aminoglycoside-modifying enzymes which play a major role in conferring resistance to aminoglycosides; (iv) target site modifications; and (v) reduced permeability through decreasing the expression of some porins (Lee et al. 2017). The combination of multiple resistance mechanisms in *A. baumannii* has limited the number of antibiotic classes available to treat infections caused by this pathogen (Banoub et al. 2021).

A significant asset in the arsenal of *A. baumannii* is its remarkable genetic plasticity, which enables swift genetic rearrangements and mutations, along with the incorporation of foreign determinants acquired through the horizontal transmission of mobile genetic elements (MGEs) (Howard et al. 2012). Insertion sequences are regarded as crucial MGEs in influencing bacterial genomes and, consequently, evolution (Elbehiry et al. 2023).

## Phage therapy as an alternative treatment for MDR *A. baumannii*

Owing to the increased prevalence of *A. baumannii* resistance against existing antibiotics, there is a heightened urge to develop new strategies for preventing and treating *A. baumannii* infections. A promising non-antibiotic alternative for treating MDR *A. baumannii* infections is the use of phage therapy (Weinberg et al. 2020). Phage therapy is expected to gain popularity in the twenty-first century because, unlike antibiotics that affect a broad range of bacteria, bacteriophages are highly specific to their host bacteria, while leaving beneficial microbiota unharmed (García-Quintanilla et al. 2013). For almost a century, scientists have researched the use of bacteriophages to treat infections caused by bacteria. Phages lyse target bacterial cells, but they do not affect mammalian cells. Bacterial species are targeted by bacteriophages, which bind to receptors on the cell surfaces of bacteria to inject their genetic material, ultimately causing cell lysis during the lytic phase (Vrancianu et al. 2020). Phages were used in a recent study to treat a patient with necrotizing pancreatitis that became exacerbated by an MDR *A. baumannii* infection. After receiving two distinct phage cocktails intravenously and through intra-abdominal drains, the infection was successfully resolved and the patient experienced full clinical recovery (Singha et al. 2024).

## Bacteriophage characteristics

Phages are obligate intracellular parasites that can multiply inside bacteria using some or all of their biosynthetic machinery, they are much smaller than bacteria and can be found in soil, feces, or sewage (Clokie et al. 2011; Hatfull and Hendrix 2011). The size of the nucleic acid depends on the phage, the simplest phages have only nucleic acids that are enough to code for an average 3–5 size gene, while the more complex phages may have nucleic acids that code for over 100 gene products (Clokie et al. 2011). Phages commonly consist of a capsid (head), genetic material, neck, and tail. A few phages have lipid envelopes, but most are naked (Madigan et al. 2021). The head contains and protects the genetic material of the virus, which in turn can either be DNA or RNA, single-stranded or double-stranded, circular or linear (Clokie et al. 2011; Hatfull and Hendrix 2011). The tails of some bacteriophages are contractile and function in DNA entry into the host, while other phages have flexible, non-contractile tails (Madigan et al. 2021). The tail spikes at the end of the tail determine the host specificity of the phage since they mediate the phage’s attachment to specific receptors on the bacterial host surface, including bacterial lipopolysaccharides, teichoic acids, flagella, pili, or porin transmembrane proteins (Madigan et al. 2021; Taslem Mourosi et al. 2022).

The initial classification of bacteriophages was based on their morphology, for instance filamentous, tailed, or icosahedral phages. They were further classified according to their genetic material as single-stranded, double-stranded, DNA, or RNA (Ackermann 2009). The current phage classification continues to utilize morphology but in addition to other properties such as genome, proteome, and phylogeny (Tolstoy et al. 2018). In 2023, the bacterial viruses Subcommittee of the International Committee on Taxonomy of Viruses (ICTV) updated the classification of phages by eliminating the morphology-based families *Siphoviridae*, *Myoviridae*, and *Podoviridae*, and ratified the order *Caudovirales* to the class *Caudoviricetes*. This change was made to reflect their shared evolutionary histories (Turner et al. 2023). According to ICTV, bacteriophages now belong to 67 viral families (https://ictv.global/report/chapter/information/information/diagrams, accessed 9 Jan 2025).

## Bacteriophage life cycle: lytic or lysogenic?

**Fig. 1:**
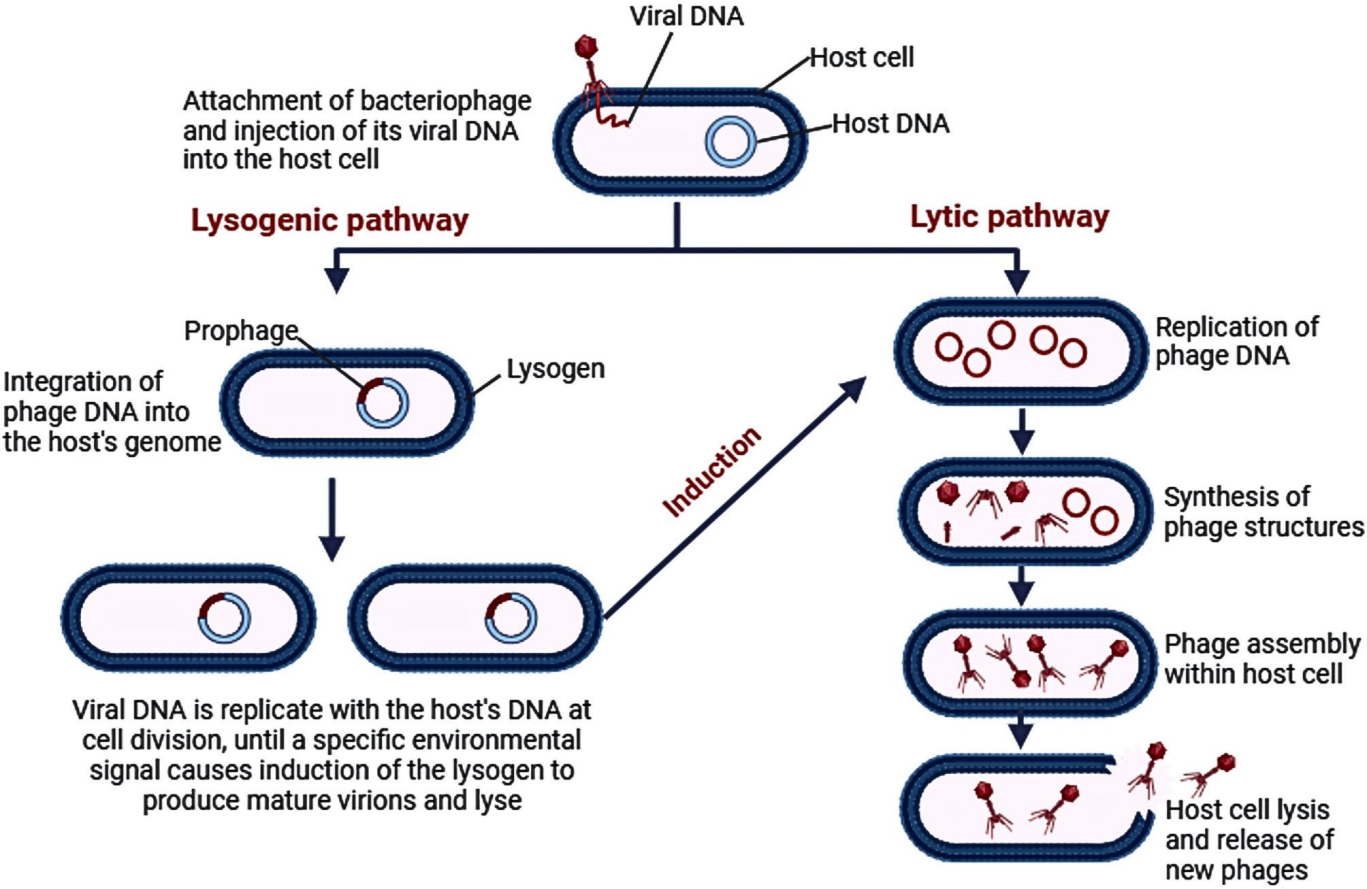
The difference between the lytic and lysogenic life cycles of bacteriophages. Image created via Biorender (https://BioRender.com)

Phages can be classified according to their life cycles and action on hosts into virulent and temperate phages (Maciejewska et al. 2018). Phages may follow a lytic pathway leading to the lysis of their hosts after infection, in such cases, they are considered “virulent phages”. Alternatively, they may follow a lysogenic pathway where the phage integrates its genetic material, called a prophage, within the host’s genome, leading to the replication of the prophage in synchrony with the host’s genome without killing the host, these phages are called “temperate phages” (Strathdee et al. 2023). A lysogenic cycle occurs due to the expression of a phage-encoded repressor protein that controls genes on the prophage, as well as prevents gene expression by any identical or closely related phage that attempts to infect the same host cell. This results in lysogens—host cells harboring a temperate phage—being resistant to infection by the same type of phage (Madigan et al. 2021). Lysogens can sometimes survive with additional antimicrobial resistance genes, virulence genes, and toxins newly gained from the invasive phage. It is important to mention that occasionally, temperate phages are induced to enter a lytic cycle. If the synthesis of the phage repressor is prevented or if it becomes inactivated, the prophage is induced, the host cell is lysed, and new virions are released (Strathdee et al. 2023; Tu et al. 2023). Since lysogeny can enhance bacterial resistance and virulence, only lytic phages should be used for bacteriophage therapy (Kakasis and Panitsa 2019). The difference between the lytic and lysogenic life cycles of bacteriophages is depicted in Fig. 1. An example of antibiotic resistance acquired by an *A. baumannii* strain is the transduction of the *bla*_NDM-1_ gene that codes for carbapenemase enzyme obtained from another strain of the same species, which resulted in its carbapenem resistance in a study done by Krahn T. et al. This study explored the impact of how *A. baumannii* strains could transfer antibiotic resistance genes to one another after the activation of a strain’s prophages because of its infection with a temperate phage (Krahn et al. 2016). It is for this reason that virulent phages are the ones studied for the treatment of MDR bacterial infections, due to their ability to destroy bacterial cells, in contrast to temperate phages which contribute to the high rate of host cells’ survival (Strathdee et al. 2023).

## Phage lysis

Double-stranded virulent phages lyse their host through the destruction of the host cell wall via certain phage products primarily: endolysins, holin and spanins (Cahill and Young 2019). These proteins form the endolysin-holin system which starts with the embedded holin locally depolarizing the host’s cell membrane, forming holes within it, followed by the escape of accumulated lysin from the cytoplasm towards the peptidoglycan component of the cell wall where it degrades it and results in its osmotic imbalance, which leads to the host’s death (Tu et al. 2023; Vázquez et al. 2021).

The cell lysis can be done by other mechanisms. The use of phage products directly—known as “ enzybiotic therapy”—particularly the lysins system, provides a new potential alternative to phage therapy considering the increased specificity and reduced risk of resistance compared to both phage therapy and antibiotics (Vázquez et al. 2021). Multiple studies are currently discussing the use of phage lysins for the treatment of MDR infections, such as PlyF307 phage lysin whose C-terminal peptide P307 was engineered to provide high therapeutic activity against *A. baumannii* infections. These phage products show a promising future to overcome the MDR *A. baumannii* crisis, especially since they can be modified artificially to maximize their bactericidal abilities (Thandar et al. 2016).

## Acquisition and characterization of bacteriophages

Phages are plentiful and can be easily obtained without the need for complex experimental manipulation or specialized equipment, making them accessible for both research and clinical applications. The process of isolating bacteriophages is straightforward and cost-effective, facilitating the rapid formulation of personalized treatments in industrial or clinical settings (Dempsey and Corr 2022). Bacteriophages are found in a variety of settings, including the ocean and the microbial environment inside microorganisms, and are thought to be a component of a complex ecosystem (Elois et al. 2023). Furthermore, the biome has a direct impact on the type and quantity of phages found in soils. The type of soil, its chemical makeup, and the quantity of bacteria present all influence it. Due to the great prevalence of these viruses in human feces, the human gut is also thought to represent a phage habitat. The primary sources for isolation of bacteriophages include water samples from lakes, rivers, sewage treatment facilities, and hospital sewage as phages are mainly found wherever the bacterial host exists (Elois et al. 2023; Tu et al. 2023). Because hospital sewage water contains a large amount of both organic and non-organic material necessary for bacterial host proliferation, it is regarded as one of the best media for phage growth (Elois et al. 2023; Tu et al. 2023).

Although bacteriophage discovery techniques vary depending on laboratory preferences, phage extraction typically involves four primary steps: isolation, purification, propagation, and characterization (Hitchcock et al. 2023). There are two main methods for the isolation of phages from environmental samples: direct inoculation, and enrichment. Direct inoculation is done by directly plating environmental samples with an indicator bacterial host strain to detect the formation of plaques or clear spots indicating the presence of the phage. For this method to be successful, a relatively high concentration of phages should be present in the environmental sample since only a small volume of the sample can be used on a single plate (about 1 ml for plaque assay or 10 µl for spot test) (Hyman 2019). Direct plating has been successfully used by Elshamy et al. to isolate phages against carbapenem-resistant *A. baumannii* (CRAB) from hospital sewage samples using plaque assay (Elshamy et al. 2023). When phages are difficult to isolate directly or when their concentration in the environmental sample is low, enrichment procedures are often required (Clokie and Kropinski 2009).

Phage characterization includes identifying morphological, biological, and genomic features of the phage. Morphological characteristics are commonly identified by transmission electron microscopy (Elshamy et al. 2023; Yuan et al. 2021). Biological characteristics and stability studies include determining the multiplicity of infection (MOI)– the ratio of viral particles to host cell (Wei et al. 2023), growth curve (Wu et al. 2019), phage stability at different temperatures and pH values (Cha et al. 2018; Kropinski et al. 2009a), host range (Kutter 2009), and in some cases the chloroform tolerance of the phage to detect the absence or presence of lipid components in the tail or capsid (Wei et al. 2023). Genomic characterization involves DNA extraction, sequencing and genomic analysis of the phage genome. An online bacteriophage database called “PhageScope” offers comprehensive phage annotations, including taxonomic annotation, structural and functional annotation, completeness assessment, phenotype annotation, and phage genome comparison. Currently, this database harbors the sequences and metadata of 873,718 phages, 2,568 of which infect *A. baumannii* as a host bacterium (https://phagescope.deepomics.org/database/phage, accessed 11 Jan 2025) (Wang et al. 2023).

## Phage nomenclature

The most common method of bacteriophage naming is done using a three-part name that consists of the genus name of the bacterial host, the word "phage," and a unique identifier. Information regarding the phage's host and morphology are examples of unique identifiers. For example, the name Acinetobacter phage vB_AbaM-SW8 denotes a virus of bacteria, infecting *Acinetobacter baumannii*, with myovirus morphology, named SW8. The phage's common name is typically the last portion of the name (SW8) (Kropinski et al. 2009b; Adriaenssens and Brister 2017). By searching the Phage Name Check website (http://www.phage.org/phage_name_check.html#calculator), the suggested names are compared to those that are already in use in the field to prevent using pre-existing phage names.

## In vitro studies against MDR *A. baumannii*

In 2020, A study by Kim et al. assessed the antimicrobial activity of LysSS, a novel phage-derived endolysin against MDR *A. baumannii* and *P. aeruginosa*. Unlike antibiotics, endolysins specifically target bacterial peptidoglycan, leading to cell lysis with minimal impact on beneficial microbiota. The research provided comprehensive insights into LysSS’s molecular characteristics, in vitro antimicrobial efficacy, and potential therapeutic applications, thereby contributing to the development of innovative solutions for combating MDR bacterial infections (Kim et al. 2020). A study by Luo et al. evaluated a combination strategy through bactericidal synergism between Phage YC#06 and antibiotics to target MDR *A. baumannii *in vitro and in vivo. The in vitro investigations of this study showed phage-antibiotic synergy against MDR *A. baumannii* with chloramphenicol, minocycline, imipenem, and cefotaxime (Luo et al. 2022). This approach not only enhances bacterial eradication but also mitigates the development of antibiotic and phage resistance, offering a sustainable solution for managing difficult-to-treat infections (Kebriaei et al. 2020; Gu Liu et al. 2020).

In 2024, the one-step growth curve in a study by Ndiaye et al. using Phage vAbaIN10 against CRAB showed that vAbaIN10 had a latency period of 25 min and a burst size of approximately 4.78 × 10^3^ phages per infected bacterial cell. The absence of tRNA, mtRNA, clustered regularly interspaced short palindromic repeats (CRISPR), virulence factors, and antibiotic-resistance genes in the genome suggested that it may be used in phage therapy (Ndiaye et al. 2024). In another study by Rothong et al., 3 antimicrobial peptides were designed from the endolysin of *A. baumannii* bacteriophages vB_AbaM_PhT2 and vB_AbaAut_ChT04 and evaluated against MDR and XDR *A. baumannii*, results showed that when combined with colistin, the three peptides had significant synergistic effects with minimum inhibitory concentrations (MICs) ranging from 156.25 to 312.5 µg/ml (Rothong et al. 2024). A lytic Phage HZY2308 was evaluated against an MDR *A. baumannii* AB48 isolate in an in vitro study in China, when used in combination with tigecycline, phage HZY2308 demonstrated synergistic antibacterial activity and decreased the MDR *A. baumannii* AB48 isolate's capacity to form biofilms. An *A. baumannii* resistant to HZY2308 was developed by natural selection and showed increased susceptibility to cefepime, gentamicin, amikacin, and tobramycin, suggesting an evolutionary trade-off (Wang et al. 2024b). In an in vitro and pre-clinical study in China testing K3-lytic phage P1068 targeting the K3 capsule type against CRAB. In vitro antimicrobial experiments showed that P1068 was effective against *A. baumannii* in both biofilm and planktonic stages. In mice models of intraperitoneal infection, the P1068 phage protected animals against *A. baumannii* infection and significantly reduced bacterial loads in the brain, blood, lung, spleen, and liver as compared to controls. The findings suggested that this phage could be used to treat *A. baumannii* infections that form biofilms and are carbapenem-resistant (Zheng et al. 2024). A novel lytic phage vB_AbaAut_ChT04 was tested against MDR *A. baumannii* by Leungtongkam et al., the isolated phage showed stable physicochemical properties, and a gene coding for endolysin was isolated. The produced antimicrobial peptide (PLysChT04) showed significant antimicrobial activity against MDR *A. baumannii* clinical isolates (Leungtongkam et al. 2023).

Margulieux et al. studied the broad host range *Acinetobacter baumannii* phage EAb13 which was isolated from sewage, this phage showed a remarkable action against MDR *A. baumannii* (Margulieux et al. 2023). Erol et al. attempted to study the in vitro antibiofilm activity of bacteriophages C2 phage, K3 phage and phage cocktail (C2 + K3 phage) in combination with colistin. They found that combining phage and colistin increased the removal of biofilms of MDR *A. baumannii* clinical isolates and gave new information on how to treat infections linked to biofilms that are generated by antibiotic-resistant bacteria (Erol et al. 2023). In India, Vashisth et al. studied the virulent phage φAB182 which demonstrated a strong synergistic effect with colistin against the biofilm of MDR *A. baumannii* clinical isolates, followed by polymixin B, ceftazidime, and cefotaxime. The synergistic effect was confirmed by time-kill kinetics (Vashisth et al. 2022). A novel phage lysin Abp013 was evaluated in vitro against MDR *A. baumannii* clinical isolates by Chu et al. in Singapore, Abp013 showed notable lytic efficacy against MDR *A. baumannii* isolates and was able to reach and destroy the bacterial cells living in the biofilm (Chu et al. 2022). In Egypt, Abdelkader et al. studied DpoMK34, the specific capsule depolymerase of phage PMK34, against *A. baumannii* clinical isolate MK34. The results of functional characterization showed that the mesophilic enzyme DpoMK34 was specific to the capsule of *A. baumannii* MK34 and was active up to 50°C throughout a wide pH range (4 to 11). Phage adsorption dramatically dropped from 95 to 9% after five minutes as a result of the *A. baumannii* MK34 capsule's enzymatic breakdown (Abdelkader et al. 2022). The important highlights of in vitro studies using phages against MDR *A. baumannii* clinical isolates are summarized in Table 1.

**Table 1:** In vitro studies using phages against MDR *A. baumannii clinical* isolates

Used phage(s)	Tested bacterium	Nature of study	Main findings	Date of study/source or location of the study	References/Source
Phage vAbaIN10	Carbapenem resistant *A. baumannii*	In vitro	According to the one-step growth curve, vAbaIN10 had a burst size of roughly 4.78 × 10^3^ phages per infected bacterial cell and a latency period of 25 min. Its possible application in phage therapy is indicated by the absence of tRNA, mtRNA, CRISPR, virulence factors, and antibiotic-resistance genes in the genome	2024/Dakar, Senegal	Ndiaye et al. (2024)
Three antimicrobial peptides were designed from the endolysin of *A. baumannii* bacteriophages vB_AbaM_PhT2 and vB_AbaAut_ChT04	MDR and XDR *A. baumannii, E. coli, K. pneumoniae, P. aeruginosa, S. aureus*, and *B. subtilis*	In vitro	The three peptides showed substantial synergistic effects with colistin, with MICs ranging from 156.25 to 312.5 µg/ml	2024/Thailand	Rothong et al. (2024)
A lytic Phage HZY2308	MDR *A. baumannii* AB48	In vitro	Phage HZY2308 reduced the MDR *A. baumannii* AB48 isolate's ability to build biofilms and shown a synergistic antibacterial activity when coupled with tigecycline. Through natural selection, an *A. baumannii* that is resistant to HZY2308 was developed and showed increased susceptibility to cefepime, gentamicin, amikacin, and tobramycin, indicating an evolutionary trade-off	2024/China	Wang et al. (2024b)
K3-lytic phage P1068 targeting the K3 capsule type	Carbapenem-resistant *A. baumannii*	In vitro and preclinical (animal model)	P1068 demonstrated antibacterial effectiveness against *A. baumannii* in both biofilm and planktonic stages, according to in vitro antimicrobial studies. When compared to controls, the P1068 phage dramatically decreased bacterial burdens in the brain, blood, lung, spleen, and liver while also protecting animals against *A. baumannii* infection in mouse models of intraperitoneal infection. According to the results, this phage may be used to treat infections caused by *A. baumannii* that build biofilms and are resistant to carbapenem	2024/China	Zheng et al. (2024)
A novel lytic phage vB_AbaAut_ChT04	MDR *A. baumannii* clinical isolate	In vitro	A novel lytic phage vB_AbaAut_ChT04 was isolated, and it showed stable physicochemical properties. A gene coded for endolysin was isolated and the produced antimicrobial peptide (PLysChT04) showed significant antimicrobial activities against MDR *A. baumannii* clinical isolates	2023/Thailand	Leungtongkam et al. (2023)
Phage EAb13	MDR *A. baumannii* clinical isolate	In vitro	The genome of the lytic phage EAb13, which was isolated from sewage and has extensive action against *A. baumannii* that is resistant to multiple drugs, is described. EAb13 is a siphovirus that has not been classified. Its genome is 82,411 bp long, contains 126 protein-coding sequences, 1 tRNA, 2,177 bp long direct terminal repeats, and 40.15% GC	2023/USA	Margulieux et al. (2023)
bacteriophage C2 phage, K3 phage and phage cocktail (C2 + K3 phage) in combination with colistin	MDR *A. baumannii* clinical isolates (n = 24(	In vitro (anti biofilm activity)	Combining phage and colistin increased the removal of biofilms and gave new information on how to treat infections linked to biofilms that are generated by bacteria that are resistant to antibiotics	2023/Turkey	Erol et al. (2023)
A virulent phage (φAB182)	MDR *A. baumannii* clinical isolates	In vitro (anti biofilm activity)	Phage φAB182 demonstrated the strongest synergy with colistin when antibiotics were present, followed by polymixin B, ceftazidime, and cefotaxime. Time-kill kinetics further confirmed this synergistic effect. Phage φAB182 worked in concert with colistin, polymixin B, ceftazidime, and cefotaxime to eradicate the biofilms that *A. baumannii* had created	2023/India	Vashisth et al. (2022)
A novel phage lysin Abp013	MDR *A. baumannii* clinical isolates	In vitro	Significant lytic activity was demonstrated by Abp013 against isolates of *A. baumannii* that were resistant to many drugs. Interestingly, Abp013 could withstand up to 10% of human serum and can reach and destroy the bacterial cells living in the biofilm	2022/Singapore	Chu et al. (2022)
DpoMK34– the specific capsule depolymerase of Phage PMK34	*A. baumannii* clinical isolate MK34	In vitro and in silico	DpoMK34’s functional characterization showed that it is a mesophilic enzyme that is unique to the capsule of *A. baumannii* MK34 and active up to 50°C over a broad pH range (4 to 11). After five minutes, phage adsorption significantly decreases from 95 to 9% due to enzymatic breakdown of the *A. baumannii* MK34 capsule. DpoMK34 makes *A. baumannii* MK34 completely vulnerable to serum death in a serum concentration-dependent manner, despite the fact that it lacks inherent antibacterial activity	2022/Egypt	Abdelkader et al. (2022)

Clustered regularly interspaced short palindromic repeats; MDR, multidrug-resistant; XDR, extensively drug-resistant

## Phage therapy studies against *A. baumannii* in animal models

Phage treatment shows potential as an antibiotic substitute during the growing trend of antibiotic resistance. Animal models are used in most properly designed phage treatment experiments. It is essential to comprehend the achievements of these models and figure out how to enhance them before moving on with human clinical trials (Penziner et al. 2021). The following are in-vivo models of phage therapy that have shown promising findings against *Acinetobacter* infections. Kusradze et al. aimed to illustrate phage therapy as a non-toxic, safe, and possible substitute for antibiotic treatment. In their control group, phage application showed no toxic impact on wounded rats, and no signs of aggression. The phage, vB-GEC Ab-M-G7, was used topically in wound infections inoculated by *A. baumannii* T-10 and G7. It significantly decreased the bacterial load from treated animals, and all signs of infection (red, swelling, purulent lesion) that were initially present have vanished (Kusradze et al. 2016).

In another study by Shivaswamy et al., the ability of bacteriophage to treat wound infection in uncontrolled diabetic rats due to MDR *A. baumannii* was evaluated. Within 5 groups of rats studied, two of which, colistin and phage treated, had comparable results. The treatments were given after 48 h of infection. After six days, no bacteria were isolated from the abscess in the phage-challenged group, suggesting that the *Acinetobacter* phage had eliminated all the MDR *A. baumannii* that was present in the abscess. But in the group that was given colistin, bacteria were still present in the abscess till day 14. Phages considerably shortened the period of epithelialization and raised the percentage of wound contraction in the rat excision wound model (Shivaswamy et al. 2015). In a mouse model with an MDR *A. baumannii* nasal infection, the therapeutic effectiveness of a phage cocktail including two newly discovered phages (PBAB08 and PBAB25) and additional phages was assessed. In the seven days following infection, mice given the phage cocktail had a 2.3-fold increased survival rate compared to untreated mice. Furthermore, a 1/100 decrease in the bacterial load was noted in the lungs of the mice after the phage cocktail treatment. Additionally, the inflammatory reactions of mice given the phage mixture intraperitoneally, intranasally, or orally were studied. There was very little increase in serum cytokine in all methods of injection (Cha et al. 2018).

A similar study on mice was conducted by Cyclophosphamide-induced immunosuppression which developed into *A. baumannii*-mediated pneumonia. The outcomes of this research showed that the therapeutic effectiveness of using a virulent bacteriophage to treat *A. baumannii* pneumonia in mice was dose- and time-dependent. Consequently, making them the key factors affecting the efficacy (Wang et al. 2016). The administration of phage therapy 1h-post infection reduced the inflammatory response and restored the mouse lung tissue to a healthy state when seen by microcomputed tomography, also as demonstrated by the lung tissue's H&E stain (Wang et al. 2016).

The results of 2 mice models of inoculation by *A. baumannii* were studied by Lood et al. The first model mimicked an infection by contaminated catheter via inserting a 3 cm catheter section consisting of 2-day preformed biofilm subcutaneously, then treated with phage lysins PlyF307 24 h later. The catheter was removed, and residual bacteria were counted to find a 2-log decrease in bacterial viability. A possible approach is suggested in which human patients with infected implants can be treated without removing them surgically as there was accompanied by significant degradation of the extracellular polymeric matrix (Lood et al. 2015). The second was a sepsis model where mice were injected intraperitoneally with 10^8^ CFU bacteria and treated with the phage or a buffer 2 h later. 90% of the mice treated with buffer died within a day or two, whereas mice treated with PlyF307 had a 50% higher survival rate (Lood et al. 2015).

Bacteriophages were employed to combat CRAB infections, as detailed in a study by Jeon et al. The study utilized two in-vivo models: mice and *Galleria mellonella*, to simulate acute pneumonia (Jeon et al. 2019). Phage Bϕ-R2096 notably increased survival rates in both *G. mellonella* larvae (from 0 to 50% at 24 h) and mice (from 30% at MOI = 0.1 to 100% at MOI = 10 over 12 days) following CRAB infection. In the mouse pneumonia model, phage Bϕ-R2096 significantly reduced histological lung damage, with bacterial clearance observed by day 3 post-infection. Importantly, in vivo experiments on phage-treated groups showed no mortality or adverse effects. These findings strongly indicated that the virulent phage (Bϕ-R2096) targeting *A. baumannii* could potentially serve as an alternative to antibiotics for treating CRAB infections (Jeon et al. 2019).

Phage-derived polysaccharide depolymerase potentiates, Dpo71, was evaluated against MDR *A. baumannii* causing pneumonia. The animal model results showed that Dpo71 enhanced ceftazidime's effectiveness against *A. baumannii* pneumonia via low-serum-dependent mechanisms (Wang et al. 2024a). In another study, genomic analysis of a novel lytic saclayvirus phage vB_AbaM_P1 was done by Li et al. The use of phages decreased the rat mortality from *A. baumannii* as well as the bacterial burden in the lungs. Histological investigations revealed fewer immune cells in the lung tissue. The substantial effect of phage vB_AbaM_P1 in preventing *A. baumannii* infection was demonstrated by the decrease in oxidative stress in lung tissue and cytokine levels in serum (Li et al. 2024a).

A study from Korea studied the effect of Phage vB_AbaSi_W9 against *A. baumannii* ATCC17978 as a host strain and 29 CRAB isolates. When tigecycline and rifampicin were combined, Phage vB_AbaSi_W9 and antibiotics demonstrated a significant synergistic impact, suggesting that they could be utilized as a combination therapy to treat CRAB infections (Choi et al. 2024). In a different study from China, the in vivo efficacy of phage cocktails was tested against CRAB in the rat pneumonia model. The therapeutic efficacy of the phages was validated by transcriptomic analysis of rat lung tissue during phage treatment, which showed notable changes in the immune system. The findings of this study demonstrate the potential of phages for further development as therapy and offer compelling information on immune system dynamics throughout treatment (Li et al. 2024b).

In a study evaluating the efficacy of phage therapy against PDR *A. baumannii*, when the lytic phage ФAb4B was administered in combination with ciprofloxacin, 91% of the mice in the bacteremia animal model were saved, which was superior to using phage alone (67%) (Wang et al. 2024c). According to the in vivo results of a recent study on CRAB, the survival rate of mice and *Galleria mellonella* infected with CRAB was significantly increased by phage Ab_WF01 after seven days. Moreover, phage Ab_WF01 considerably reduced the inflammatory response and enhanced histological damage and bacterial clearance in infected tissue organs after day 3 post-infection in the mouse CRAB infection model (Wang et al. 2024d).

The phage vB_AbaM-IME-AB2 was combined with colistin and formulated as a hydrogel, the effect of which on MDR *A. baumannii* infected mouse wound model was studied. The hydrogel formulation's antibacterial efficacy significantly reduced the MDR *A. baumannii* load by 4.65 log by mixing colistin with the vB_AbaM-IME-AB2 phage, suggesting its appropriateness for treating *A. baumannii* skin infections (Mukhopadhyay et al. 2024). In a study by Ilomuanya et al., phages ɸAB140 and ɸAB150 alone and encapsulated in combination within a chitosan microparticle were evaluated against MDR *A. baumannii* in an infected mouse wound model. The lytic activity against MDR *A. baumannii* was shown using microparticle carrier technology. In vivo, a significant reduction in wound size was seen, with the most apparent reduction occurring in encapsulated C2 microparticle hydrogel (Ilomuanya et al. 2022). A study from Iraq by Jasim et al. researched the effect of phage cocktail on XDR, and PDR *A. baumannii* in an infected mouse model. The phage therapy assessed in this study was able to effectively lyse most XDR and PDR bacteria both in vitro and in vivo, and it was shown that the phage cocktail was more effective than single-phage preparations in treating *A. baumannii* (Jasim et al. 2018). In a study using a novel temperate phage vB_AbaM_ABMM1 infecting *A. baumannii* as a host strain in an infected zebrafish model, despite its ability to integrate into the host genome, the high MOI of ABMM1 effectively killed the host bacterial cells and reduced the mortality rate of bacterial infection in the zebrafish model. These findings imply that ABMM1 might be employed as an alternative treatment for infections caused by *A. baumannii* (Mardiana et al. 2023). The important highlights of in vivo studies using phages against MDR *A. baumannii* clinical isolates are summarized in Table 2.

**Table 2:** In vivo studies using phages against MDR *A. baumannii clinical* isolates

Used phage(s)	Tested bacterium	Nature of study	Main findings	Date of study/source or location of the study	Reference/Source
Inhaled therapy using the Phage BA3	Extensively drug-resistant *A. baumannii*	in vivo and case report	Human blood contains the inhaled phages, which have a tendency to gather in the intestines. Additionally, during the course of the phage treatment, significant alterations in the gut microbiome were noted	2024/China	Qu et al. (2024)
Phage-derived polysaccharide depolymerase potentiates, Dpo71	MDR *A. baumannii* causing Pneumonia	Preclinical (animal model)	Through low-serum-dependent processes, phage-derived polysaccharide depolymerase Dpo71 enhanced ceftazidime's effectiveness against *A. baumannii* pneumonia	2024/China	Wang et al. (2024a)
A lytic saclayvirus phage vB_AbaM_P1	MDR *A. baumannii* clinical isolate	Preclinical (animal model)	Both the bacterial burden in the lungs and the rat mortality from *A. baumannii* were reduced because of the use of phages. There were fewer immune cells in the lung tissue, according to histologic analysis. The reduction of oxidative stress in lung tissue and cytokine levels in serum demonstrated the significant effect of phage vB_AbaM_P1 in preventing *A. baumannii* infection	2024/China	Li et al. (2024a)
Phage vB_AbaSi_W9	*A. baumannii* ATCC17978 as a host strain and 29 carbapenem-resistant *A. baumannii*	preclinical	An in vivo mouse infection model was used to confirm the phage and rifampicin's synergistic action in vitro. Phage vB_AbaSi_W9 and antibiotics showed a strong synergistic impact in experiments when tigecycline and rifampicin were used together, indicating that they could be employed as a combined therapy to treat CRAB infections	2024/ Korea	Choi et al. (2024)
Phage cocktail	Carbapenem-resistant *A. baumannii*	Preclinical (rat pneumonia model)	Transcriptomic analyses of rat lung tissue during phage treatment demonstrated significant alterations in the immune system, and the phages' therapeutic effectiveness was confirmed. The results of our study provide strong evidence on immune system dynamics during the course of treatment and highlight the potential of phages for future development as a therapeutic approach	2024/China	Li et al. (2024b)
Lytic Phage ФAb4B	PDR *A.baumannii* YQ4	Preclinical (Bacteremia animal model)	When phage and ciprofloxacin were administered together, 91% of the mice were saved, which was better than when phage was used alone (67%). The combinatorial treatment's effectiveness was unaffected by the phage dosage	2024/China	Wang et al. (2024c)
A novel phage, Ab_WF01	carbapenem-resistant *A. baumannii*	Preclinical (Infected mouse model)	According to the in vivo findings, phage Ab_WF01 considerably raised the survival rate of mice and *Galleria mellonella* infected with CRAB after seven days. Furthermore, in the mouse CRAB infection model, phage Ab_WF01 significantly improved histological damage and bacterial clearance in infected tissue organs after day 3 post-infection, reducing the inflammatory response	2024/China	Wang et al. (2024d)
vB_AbaM-IME-AB2 phage combined with colistin	MDR *A. baumannii* clinical isolate	Preclinical (Infected mouse wound model)	By combining colistin and the vB_AbaM-IME-AB2 phage, the hydrogel formulation's antibacterial effectiveness dramatically decreased the MDR A. baumannii burden by 4.65 log, indicating that it is suitable for managing A. baumannii skin infections	2023/China	Mukhopadhyay et al. (2024)
Phage øFG02 alone or in combination with ceftazidime	MDR *A. baumannii* clinical isolates	Preclinical (Infected mice model)	*A. baumannii* AB900's in vivo development into a capsule-deficient, phage-resistant phenotype that is resensitized to ceftazidime is consistently driven by øFG02. This technique demonstrates how phage therapy can be used to target *A. baumannii* and restore antibiotic activity in a clinical setting	2022/Australia	Gordillo Altamirano et al. (2022)
phages ɸAB140 and ɸAB150 alone, in combination encapsulated within a chitosan microparticle	MDR *A. baumannii* clinical isolates	Preclinical (Infected mouse wound model)	Utilizing microparticle carrier technology, the lytic activity against MDR *A. baumannii* was demonstrated. Significant wound size reduction was seen in vivo, with encapsulated C2 microparticle hydrogel exhibiting the most notable reduction	2022/ Nigeria	Ilomuanya et al. (2022)
phage cocktail	XDR, and PDR *A. baumannii*	Preclinical (Infected mouse model)	The majority of XDR and PDR bacteria could be efficiently lysed both in vitro and in vivo by the phage therapy evaluated in this investigation. It was demonstrated that the phage cocktail was more effective than single-phage preparations in treating *A. baumannii*, with a significantly lower rate of resistance to therapeutic phages	2018/Iraq	(Jasim et al. 2018)
A novel temperate phage vB_AbaM_ABMM1	*A. baumannii*	Preclinical (infected zebrafish model)	The high MOI of ABMM1 efficiently destroyed the host bacterial cells and decreased the mortality rate of bacterial infection in the zebrafish model, even though it could integrate into the host genome. These results suggest that ABMM1 may be used as a substitute therapy for *A. baumannii* infections	2023/ Taiwan	Mardiana et al. (2023)

CRAB, carbapenem-resistant *Acinetobacter baumannii*; MDR, multidrug-resistant; PDR, pandrug-resistant; XDR, extensively drug-resistant

## Clinical cases involving phage therapy against *A. baumannii*

Multiple clinical case studies illustrated successful applications of phage-antibiotic synergy in treating drug-resistant infections. One documented case involved a patient with a bone infection caused by MDR *K. pneumoniae* and XDR *A. baumannii*. Despite extensive treatment involving massive doses of antibiotics and surgical interventions, the patient’s condition persisted, emphasizing the need for alternative therapeutic solutions. The bacterial isolates were screened for phages with lytic activity. Two phages, namely ɸAbKT21phi3 and ɸKpKT21phi1, were identified (from a phage bank) to be effective against the *A. baumannii* and *K. pneumoniae* strains. They were administered to the patient intravenously along with the antibiotics meropenem and colistin. Follow-up tests conducted over eight months showed no presence of active phages or bacterial growth. This indicated that the treatment eliminated the infection and prevented further bacterial proliferation. The patient's pain also disappeared following the phage and antibiotic therapy. Overall, this study demonstrated the potential of phage therapy in treating antibiotic-resistant bone infections (Nir-Paz et al. 2019).

Qu et al. evaluated inhaled phage therapy using Phage BA3 against XDR *A. baumannii* in a case report and in vivo evaluation of the impact on the gut microbiome. Results showed that human blood contained the inhaled phages, which tended to gather in the intestines. Additionally, during the phage treatment, significant alterations in the gut microbiome were noted (Qu et al. 2024).

## Phage-antibiotic combinations as a strategy against MDR *A. baumannii*

Gordillo et al. evaluated the efficacy of phage øFG02 in conjunction with ceftazidime in a mouse model of severe *A. baumannii* infection. This study aimed to determine the in vivo bactericidal effects of the combined treatment. The primary outcomes measured were bacterial loads across four tissue sites: blood, liver, kidney, and spleen. Phage øFG02, which binds to capsular polysaccharides and induces antimicrobial resensitization in vitro, was used in this investigation to evaluate the in vivo bactericidal impact of a phage-antibiotic combination on *A. baumannii* AB900. The in vivo exposure of *A. baumannii* AB900 to øFG02 led to its resistance and evolvement to a capsule-deficient, phage-resistant phenotype that is resensitized to ceftazidime. This emphasized the clinical significance of phage therapy in targeting *A. baumannii* and restoring antibiotic efficacy. Accordingly, this investigation has highlighted the long-term benefits of combining phages with antibiotics, especially after prolonged exposure (Gordillo Altamirano et al. 2022).

## Limitations of phage therapy

Given the unprecedented global rise in antibiotic resistance, bacteriophages offer an alluring and promising possible treatment alternative. However, phage therapy is faced with some limitations. The development of phage resistance is one of the concerns when employing bacteriophages in clinical therapy. Several animal studies have shown evidence of reduced phage efficiency over time as a result of acquired phage resistance (Oechslin 2018).

**Fig. 2:**
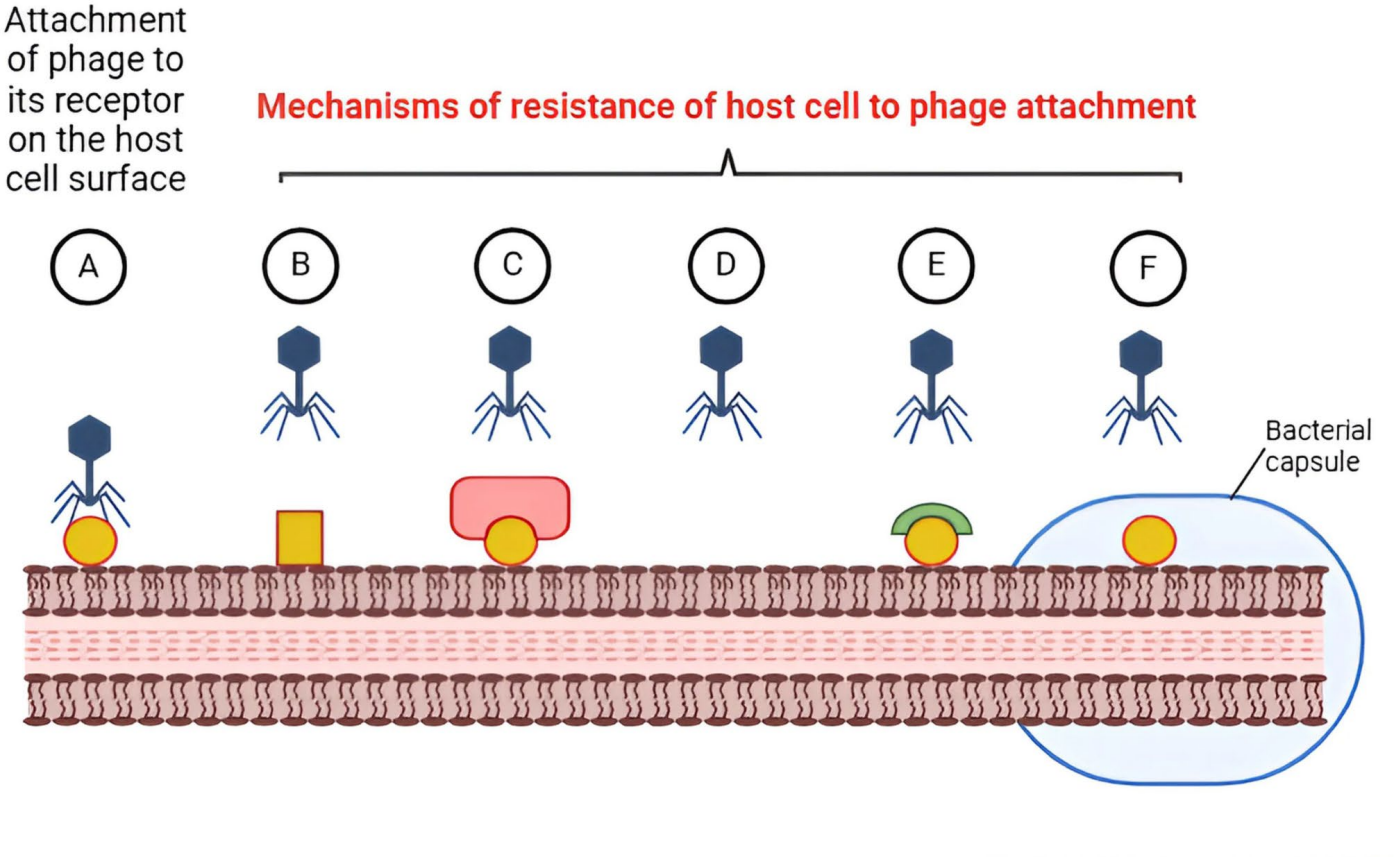
The mechanisms of bacterial resistance to phage attachment. **A** attachment of bacteriophage to its surface receptor on susceptible host bacterial cell, **B** modification of the structure of host receptor, **C** masking the surface receptors by modifying the expression of proteins that mask them, **D** downregulating or inhibiting the synthesis of the receptors, **E** competitive receptor inhibitors that occupy the active sites of the bacteriophage receptors, **F** production of extracellular polymers (e.g., capsule) which block the attachment of bacteriophages to their receptors. Image created via Biorender (https://BioRender.com)

Bacteria may develop resistance to the phage’s attachment to specific receptors on the bacterial host surface through: (i) mutation or masking their surface receptors by modifying the structure of such receptors, expression of other proteins that mask them, or via downregulating or inhibiting the synthesis of their receptors, (ii) production of extracellular polymers (e.g., capsule) which block the attachment of bacteriophages to their receptors, and (iii) competitive receptor inhibitors that occupy the active sites of the bacteriophage receptors thus interfering with its attachment (Caflisch et al. 2019; Ge et al. 2020). This resistance acquisition may be the cause of initial disease improvement at the beginning of phage therapy, followed by deterioration and infection recurrence (Aslam et al. 2020). Phages are now frequently given as “cocktails”—preparations including multiple phages with diverse modes of action—to minimize the emergence of bacterial resistance to bacteriophages (Hitchcock et al. 2023). The mechanisms of bacterial resistance to phage attachment are illustrated in Fig. 2.

Another limitation is the determination of the appropriate dose to be administered. Since phages are self-replicating pharmaceuticals, it is challenging to predict the concentration of phages at the site of infection, which may differ from the concentration of the phage when administered under a controlled environment (Payne 2000).

## Emerging and future directions in phage therapy: implications for pharmacy

Beyond current clinical trials and implementation, ongoing innovations and future directions in bacteriophage research hold transformative potential for pharmacy. These advancements include phage formulation development, personalized medicine, synthetic biology, and regulatory advancements that pharmacists must navigate in future practice. For more illustration, these advances can be summarized as follows: (i) Modern encapsulation methods, such as liposomes, alginate microbeads, and hydrogels, are improving the stability and delivery of phages (Abedon and Thomas-Abedon 2010). These formulations enable phage stability under gastrointestinal conditions for oral administration, enhancing therapeutic options for pharmacists (Malik et al. 2017). For instance, enteric-coated phage capsules have shown promising results in targeting gut infections, such as *Clostridium difficile* (Malik et al. 2017); ii) Incorporating phages into coatings for catheters, stents, or wound dressings is a growing innovation. These represent a convergence of materials science and pharmacology, offering infection-prevention solutions within hospital and community pharmacy settings (Górski et al. 2020); and (iii) The narrow host range of phages allows for the development of personalized therapies tailored to the patient’s specific bacterial infection. Pharmacists may play a critical role in collaborating with microbiologists to select and dispense individualized phage therapies. In cystic fibrosis, personalized phage cocktails are used to target *Pseudomonas aeruginosa* strains (Torres-Barceló 2018).

## Pharmacokinetics and pharmacodynamics (PK/PD)

The pharmacokinetics (PK) of phage therapy are based on the capacity of bacteriophages to self-replicate within-host bacterial cells, which results in an exponential increase in the total amount of phages at the site of infection. Even with low-dose administration, this self-replicating mechanism ensures the delivery and maintenance of effective phage concentrations at the intended target sites (Nang et al. 2023b, a). Additionally, by generating enzymes that break down the extracellular matrix, phages can disrupt bacterial biofilms, which are infamously resistant to antibiotics (Harper et al. 2014). Phage therapy PK includes absorption, distribution, metabolism, and phage elimination (Dąbrowska and Abedon 2019). Several factors affect the phage’s PK profile: (i) Phage size, shape, and cell types at the site of administration all influence phage absorption after oral and inhalation treatment, however, systemic administration results in full phage absorption to blood. (ii) The size and shape of the phages, as well as the types of cells and microbiomes, determine how phages are distributed between the blood and organs. (iii) The pH, immunological response, and microbiota all have an impact on phage metabolism. (iv) Finally, there is a negative correlation between phage size and renal elimination of the phage (Nang et al. 2023b, a).

On the other hand, phage pharmacodynamics (PD) pertains to the antibacterial activity of phage preparations. It is based mainly on the determination of MOI (Zalewska-Piątek 2023). Phages exhibit a highly specific and targeted mode of action, infecting and lysing only the target bacterial species or strain, leaving the surrounding commensal microbiota largely unaffected. This targeted approach helps to preserve the delicate balance of the host's microbiome, which is often disrupted by broad-spectrum antibiotic treatments, reducing the risk of secondary infections and associated complications (Jo et al. 2023). While the MIC is the most commonly used PD criterion for antibiotics, the absence of a standardized technique to assess the antibacterial activity of phages is a significant obstacle for phage therapy (Abedon 2022).

## Engineering phages to enhance efficacy

Synthetic biology is being used to enhance phage properties, such as broadening host ranges, evading bacterial resistance mechanisms, and improving lytic efficiency. For example, CRISPR-engineered phages are designed to precisely target antibiotic-resistant bacterial genes, such as beta-lactamase producers. Pharmacists must stay informed about these innovations, as engineered phages may be approved alongside conventional drugs for resistant infections (Lu and Collins 2007).

## Hybrid therapies

Engineered phages are also being combined with small-molecule drugs or nanoparticles for synergistic effects. Pharmacists will need to manage complex interactions and ensure proper formulation and delivery of these hybrid therapies (Schooley et al. 2017). Unlike antibiotics, phages face unique regulatory challenges due to their biological nature, variability, and the need for personalization. Pharmacists will play a critical role in understanding and adhering to regulatory frameworks, ensuring compliance in dispensing and formulation (Fauconnier 2019). Standardizing phage manufacturing processes (e.g., purification, storage, and potency testing) is crucial for therapeutic success. Ensuring the quality and stability of phage preparations will become a core responsibility for pharmacists, especially in hospital and research settings (Cooper et al. 2011). In conclusion, bacteriophage therapy is poised to revolutionize infection management, especially highly resistant pathogens like MDR *A. baumannii* which is mainly spread by ignorance of sterility measures in medical facilities and frequent movement of patients carrying the infection across different medical organizations. Bacteriophages can be obtained from the environment and can be categorized according to the mechanism of action either lysogenic or lytic pathways, which are acknowledged to be efficient by different clinical and experimental trials. A deeper understanding of *A. baumannii*'s resistance strategies against phage attacks is essential for assessing the antimicrobial efficacy of phages. Additionally, insights into the molecular mechanisms that underpin both bacterial and phage resistance can aid in the development of new phage therapies. This knowledge is crucial for addressing the growing complexity of antibiotic resistance and mitigating the impact of bacterial anti-phage strategies on phage therapy effectiveness. While phage therapy presents substantial benefits in the face of the pandemic of MDR *A. baumannii*, it also has some limitations. Consequently, pharmacists are at the forefront of this transformation. From formulation innovations to personalized medicine, engineered therapies, and regulatory challenges, phage therapy presents a multifaceted opportunity for pharmacy professionals. By being informed about emerging trends and advancing their expertise, pharmacists can lead the way in integrating phages into modern healthcare.

## Abbreviations

COPD: Chronic obstructive pulmonary disease
CRISPR: Clustered regularly interspaced short palindromic repeats
CRAB: Carbapenem-resistant *Acinetobacter baumannii*
ICTV: The International Committee on Taxonomy of Viruses
MDR: Multidrug-resistant
MGE: Mobile genetic element
MIC: Minimum inhibitory concentration
MOI: Multiplicity of infection
PD: Pharmacodynamics
PDR: Pandrug-resistant
PK: Pharmacokinetics
VAP: Ventilator-associated pneumonia
WHO: World Health Organization
XDR: Extensively drug-resistant
